# Coherence between Decomposed Components of Wrist and Finger PPG Signals by Imputing Missing Features and Resolving Ambiguous Features

**DOI:** 10.3390/s21134315

**Published:** 2021-06-24

**Authors:** Pei-Yun Tsai, Chiu-Hua Huang, Jia-Wei Guo, Yu-Chuan Li, An-Yeu Andy Wu, Hung-Ju Lin, Tzung-Dau Wang

**Affiliations:** 1 Department of Electrical Engineering, National Central University, Taoyuan 320317, Taiwan; pytsai@ee.ncu.edu.tw (P.-Y.T.); nina301221@g.ncu.edu.tw (C.-H.H.); a24250625@gmail.com (J.-W.G.); aabbcc4253@gmail.com (Y.-C.L.); 2 Graduate Institute of Electronics Engineering, National Taiwan University, Taipei 106319, Taiwan; andywu@ntu.edu.tw; 3 Cardiovascular Center, Department of Internal Medicine, Division of Cardiology, National Taiwan University Hospital, Taipei 100225, Taiwan; hungjulin@ntu.edu.tw

**Keywords:** photoplethysmography (PPG), pulse decomposition analysis (PDA), missing feature, imputation

## Abstract

Background: Feature extraction from photoplethysmography (PPG) signals is an essential step to analyze vascular and hemodynamic information. Different morphologies of PPG waveforms from different measurement sites appear. Various phenomena of missing or ambiguous features exist, which limit subsequent signal processing. Methods: The reasons that cause missing or ambiguous features of finger and wrist PPG pulses are analyzed based on the concept of component waves from pulse decomposition. Then, a systematic approach for missing-feature imputation and ambiguous-feature resolution is proposed. Results: From the experimental results, with the imputation and ambiguity resolution technique, features from 35,036 (98.7%) of 35,502 finger PPG cycles and 36307 (99.1%) of 36,652 wrist PPG cycles can be successfully identified. The extracted features became more stable and the standard deviations of their distributions were reduced. Furthermore, significant correlations up to 0.92 were shown between the finger and wrist PPG waveforms regarding the positions and widths of the third to fifth component waves. Conclusion: The proposed missing-feature imputation and ambiguous-feature resolution solve the problems encountered during PPG feature extraction and expand the feature availability for further processing. More intrinsic properties of finger and wrist PPG are revealed. The coherence between the finger and wrist PPG waveforms enhances the applicability of the wrist PPG.

## 1. Introduction

With the advances in sensors and integrated circuits, wearable devices are prosperously developed in diverse scenarios, such as applications in ambient-assisted living [1], in sports training [2], and for diagnostic support [3]. As the increase in aging population poses a challenge to the medical and social care systems globally, wearable devices for pervasive health care enabling long-term health monitoring provide an alternative solution for physiological assessment. Because cardiovascular diseases are the major cause of mortality, non-invasive sensing of cardiovascular signals, such as heart rates and blood pressures (BPs), has become a trend in biomedical consumer products.

Photoplethysmography (PPG), a low-cost optical device, can sense the blood volume changes from light intensity by either the transmission mode or reflectance mode during the cardiac cycle [4]. Furthermore, the PPG waveform contains information regarding the left ventricular ejection and the properties of the arterial tree. Hence, it has been adopted for estimations of left ventricular ejection time [5,6], BP, pulse wave velocity (PWV), and vascular age. Since the online database MIMIC II provides finger PPG, ECG, and arterial BP signals, finger PPG as well as ECG has been widely studied for BP estimation [7,8]. In addition to pulse arrival time (PAT) between ECG R peak and PPG specific feature points, such as valley, maximal slope, and systolic peak, PPG morphological features were also extracted for calculating stiffness index and augmentation index [7]. The finger PPG-based PWV estimation and vascular age estimation have been discussed [9,10]. In-depth analysis of the finger PPG features, including features from the first-order derivative PPG (FDPPG) and the second-order derivative PPG (SDPPG), was provided [11]. Correlations between finger PPG morphological features and hemodynamic parameters, such as vessel stiffness and vascular age, were shown in these works.

Wrist PPG and ECG signals were captured from smart watches in [12,13]. PTT was computed in [12] while reflective PTT, systolic period, and diastolic period were utilized in [13]. Chest PPG was measured in [14,15] and pulse arrival time (PAT) was acquired for the estimation. Compared to finger PPG, the morphological features of wrist PPG have not been addressed as much. In [16], the correlations between PWV and PAT from wrist PPG and finger PPG were investigated. Better correlation was shown for finger PPG. In [17], the authors highlighted that few studies provided comprehensive surveys about the effects of measurement sites on the PPG waveform characteristics and thus, they performed a quantitative comparison of different PPG signals. From their observations, the finger and earlobe measurement sites provided better PPG signals for feature identification and analysis. Under normal breathing, as opposed to 95% recordings from the finger site, only 67% recordings from the dorsal wrist had detectable features.

Although PPG morphological features are important to estimate BP, PWV, and vascular age, problems of missing features exist, which put a constraint on estimation algorithms or expel signals as well as subjects that can be investigated. In [11], missing features of desired local extrema in SDPPG were mentioned and an alternative equation was used for the aging index. In [18], the missing features were not included when the statistics were calculated. The authors in [19] chose the XGBoost algorithm instead of a neural network algorithm for BP estimation from PPG to combat the missing feature problem. A replacement has also been suggested for the missing dicrotic notch [9]. Recently, some authors have even considered employing the entire wave segment as the input to avoid feature engineering [20,21]. Thus, in order to increase the availability, a systematic solution to deal with missing features is necessary.

As mentioned in [17], finger PPG produced better resolvable features than wrist PPG. Thus, in this paper, we first analyze the reasons for feature disappearance and ambiguity. A missing-feature imputation and ambiguous-feature resolution technique is proposed to deal with the PPG waveforms lacking obvious morphological features or having uncertainty for feature selection. Thereafter, we aim to assess the coherence of the wrist PPG and finger PPG characteristics. The weighted pulse decomposition analysis (WPDA) [22] is applied to the synchronized finger PPG and wrist PPG signals. Then, their intrinsic properties are investigated and presented.

In the following, the signal processing flow is first illustrated in Section 2 as well as our proposed technique for missing-feature imputation and ambiguous-feature resolution. The improvement in feature extraction and the coherence of component waves from synchronized wrist PPG and finger PPG signals are described in Section 3. Further discussions are provided in Section 4. A brief conclusion is drawn in Section 5.

## 2. Methods

### 2.1. Signal Processing Flow

**Figure 1:**
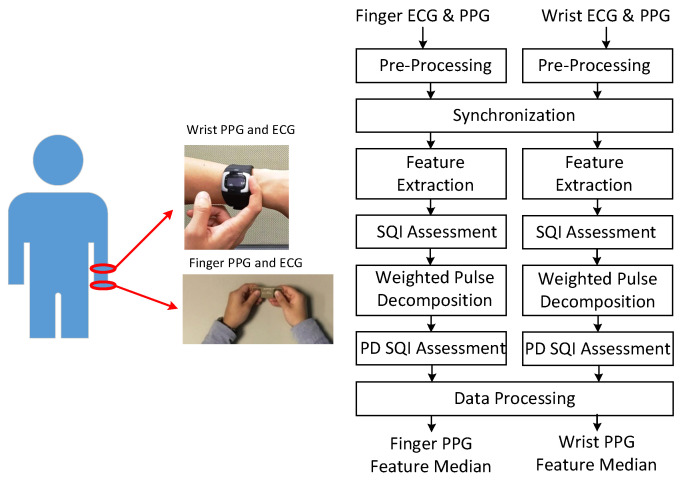
Acquisition of finger and wrist signals (**left**) and processing flow (**right**).

Figure 1 shows the signal acquisition and processing flow. In the experiment, the subjects maintained the sitting position. Finger PPG and ECG were acquired by the handheld SENSIO${}^{\mathrm{TM}}$ device on the table in the first minute of each round with a sampling rate of 512 Hz. Blood pressures were measured by the sphygmomanometer in the second minute. One-minute rest was required between adjacent rounds. During the course, the subjects wore the smart watch to obtain wrist PPG and ECG with a sampling rate of 256 Hz. The associated portions of wrist PPG and ECG signals were taken from the whole recording according to the synchronization procedure. Subjects provided 3 to 5 one-minute measurements during the experiment in the health management center and outpatient clinic, respectively.

As shown in the processing flow, these signals are first pre-processed to remove baseline wandering and 60 Hz interference. The Daubechies 8 wavelet (db8) is adopted for removing baseline wandering and the notch filter is used for eliminating the interference. Then, the finger PPG signals are down-sampled to 256 Hz. The R peaks of the wrist ECG and finger ECG signals are marked. At the synchronization stage, due to the sampling clock offset, the sequence of R–R intervals from the one-minute finger PPG signals is matched with the sequence of R–R intervals selected from a segment of three-minute wrist PPG signals by a sliding window with a shift of one R–R interval each time. After synchronization, the wrist and finger PPG cycles are aligned. Then, the PPG signals are low-pass filtered with cutoff frequency of 10 Hz and 12 Hz for heart beats below and above 80 bpm, respectively. Feature extraction and weighted pulse decomposition (PD) are performed. The qualities of extracted features and decomposed component waves are assessed by signal quality index (SQI) and PD SQI. After data processing, the medians of respective features in the one-minute signals are generated. If the correlation between finger and wrist PPG signals is examined, only the intersection of the finger and wrist PPG cycles with qualified SQI and PD SQI simultaneously is reserved.

#### 2.1.1. Feature Extraction

**Figure 2:**
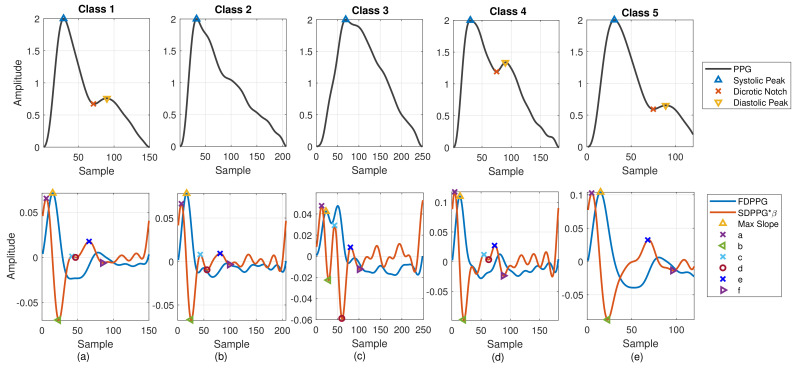
Five classes of PPG morphology features with normalized amplitude are shown. (**a**) Class 1: standard PPG, (**b**) Class 2: missing features in PPG, (**c**) Class 3: ambiguity in FDPPG, (**d**) Class 4: ambiguity in SDPPG, and (**e**) Class 5: missing features in SDPPG.

PPG morphology resembles the arterial BP waveform. Various PPG waveform features are recognized and extracted. As shown in Figure 2a, the systolic peak, dicrotic notch, and diastolic peak are defined in the PPG [4], while the first maximum is marked from FDPPG for maximal slope. Points a, b, c, d, e, and f are identified from SDPPG [23], and are assumed to be related to either forward or backward component waves. Point e is a local maximum close to the boundary of systole and diastole. Points a and c are local maxima while points b and d are local minima before e point. Point f is the first local minimum after point e.

In [24], Drawber classified PPG pulses into four types. Type I was a standard PPG containing a distinct notch. Type II and Type III had a non-developed notch. The descending slope became an almost horizontal around notch for Type II while the descending slope decreased distinguishably for Type III. Type IV showed a strong reflection in the systole. Wang et al. added Type V in [25]. No distinguishable decrease appeared in the descending slope after the systolic peak for Type V PPG. The classification focused on the PPG pulse only. In [26], seven SDPPG styles corresponding to different conditions of circulation were discussed. One style showed no c and d points in the SDPPG waveform. In this paper, considering PPG together with FDPPG and SDPPG for feature extraction, we also categorize the PPG/FDPPG/SDPPG morphological features into five classes as shown in Figure 2.

- Class 1: Standard PPG pulse contains distinguishable features including systolic peak, notch, and diastolic peak. Recognized maximum exists in FDPPG and distinguishable a to f points appear in SDPPG.
- Class 2: A single peak is shown in the PPG pulse without a recognizable notch. Usually the missing features are notch and diastolic peak in finger PPG.
- Class 3: In the FDPPG waveform, ambiguity exists for maximum selection. In the one-minute recording, sometimes, the first local maximum and the second local maximum occur before the systolic peak and become alternatively distinct depending on their strengths.
- Class 4: In the SDPPG waveform, there may be more than two maxima and two minima before point e, which was called multiple c and d points in [22]. Feature ambiguity is shown.
- Class 5: In the SDPPG waveform, the number of extrema could be less than four before e point. Usually, the missing features are c and d points.

In order to identify the extrema in the waveform, the zero-crossing points of its derivative are required. The zero-crossing points of FDPPG are used to mark systolic peak, notch, and diastolic peak in finger and wrist PPG. The first zero-crossing point of the SDPPG is searched for the maximal slope in FDPPG. The third-order derivative PPG waveform (TDPPG) is computed for finding points a to f in SDPPG. In the following, notation $n_{x}$ and $A_{n_{x}}$ represent the position of feature *x* and PPG amplitude of position $n_{x}$. The amplitude of feature *x* in the *i*th-order derivative PPG is denoted by $A_{n_{x}}^{\left(i\right)}$. After feature extraction, the signal quality index (SQI), which considers skewness [27] and R–R interval variation of the PPG pulse sequence, is then assessed to exclude PPG cycles of bad quality. Note that the PPG waveforms of Class 2 and Class 5 have missing features while the PPG waveforms of Class 3 and Class 4 have ambiguous features for selection. In Section 2.2, we describe missing-feature imputation and ambiguous-feature resolution.

#### 2.1.2. Weighted Pulse Decomposition Analysis (WPDA)

The PPG pulse consists of several forward and backward component waves. The arrival times and strengths of the component waves are regarded as clues to realize the hemodynamic state. Hence, pulse decomposition analysis is an approach to obtain further insight about the component waves. Since the Gaussian wave resembles the pulsatile wave, decomposition of the PPG pulse by Gaussian waves has been studied in [28]. Five Gaussian waves, three in the systole and two in the diastole, can fit better than four because of the rapid inflection near the onset [29]. In addition, the WPDA that emphasizes the informative portion can obtain reliable and accurate decomposition results [22]. Thus, we adopt WPDA to acquire the component waves of finger and wrist PPG waveforms for realizing their intrinsic properties.

Let $T_{s}$ be the sampling period. The Gaussian wave is described by

(1) $$G\left(t|\alpha ,\beta ,\gamma \right)=\alpha e^{\frac{{\left(t-\beta T_{s}\right)}^{2}}{2{\left(\gamma T_{s}\right)}^{2}}},$$

where α, β, and γ represent the amplitude gain, peak position, and wave width, respectively. Before pulse decomposition, the baseline of each PPG pulse is removed again and then magnitude normalization is applied. Define

(2) $$f\left(n|\Theta \right)=\sum_{i=1}^{5}G\left(nT_{s}|\alpha_{i},\beta_{i},\gamma_{i}\right)$$

where $\Theta =\left[\theta_{1}\;\;\theta_{2}\;\;\cdots \;\;\theta_{5}\right]$ and the *i*th component wave Gi is described by parameter $\theta_{i}=\left\{\alpha_{i},\beta_{i},\gamma_{i}\right\}$. Given PPG pulse P(n) and pulse length *N* samples per cycle, the WPDA tries to minimize the objective function

(3) $$\Lambda \left(\Theta \right)=\sum_{n=1}^{N}w\left(n\right){\left(P\left(n\right)-f\left(n|\Theta \right)\right)}^{2},$$

where weight w(n) is given by

(4) $$w\left(n\right)=\left\{\begin{array}{cc} w & n_{a}\leq n\leq n_{f}\\ 1 & \mathrm{else} \end{array}\right.$$

with $n_{a}$ and $n_{f}$ denoting positions of points a and f in the SDPPG waveform from feature extraction. Weight *w* is set to 80 here.

The interior point algorithm [29] is adopted for the optimization

(5) $$\hat{\Theta }=\arg\;\min_{\Theta }\Lambda \left(\Theta \right)$$

with the boundary constraints $L_{\alpha_{i}}\leq \alpha_{i}\leq U_{\alpha_{i}}$, $L_{\beta_{i}}\leq \beta_{i}\leq U_{\beta_{i}}$, and $L_{\gamma_{i}}\leq \gamma_{i}\leq U_{\gamma_{i}}$, where $L_{\alpha_{i}}\left(U_{\alpha_{i}}\right)$, $L_{\beta_{i}}\left(U_{\beta_{i}}\right)$, and $L_{\gamma_{i}}\left(U_{\gamma_{i}}\right)$ are the lower (upper) bounds of parameters $\alpha_{i}$, $\beta_{i}$, and $\gamma_{i}$, respectively. The boundary constraints are given in Table 1. Note that point $d_{n}$ denotes the zero-crossing point between point *d* and point *e* of SDPPG. If there is no zero-crossing point, the middle point between point *d* and point *e* will be used instead. Similarly, point $f_{n}$ is also the zero-crossing point between *e* and *f* and refers to their middle point with no zero-crossing point. Then, the PD SQI, which computes the mean-square error (MSE) between P(n) and f(n|Θ), is developed and qualified PPG cycles with MSE below $2\times 10^{-3}$ are reserved.

From Table 1, we see that the feature extraction plays an important role for WPDA. If a missing or ambiguous feature exists, the performance of WPDA becomes deteriorated and the decomposed component wave may not be reliable. Thus, how to deal with the five classes in Figure 2 is crucial to gain further insight into the PPG waveforms in different measurement sites.

### 2.2. Missing-Feature Imputation and Ambiguous-Feature Resolution

#### 2.2.1. Causes of Missing and Ambiguous Features

The existence of missing features and ambiguous features easily results in misjudgement for WPDA. In order to solve the problem, we need to figure out the root cause of missing features and ambiguous features. In [30], the change of the PPG waveform as well as FDPPG and SDPPG versus the change of the blood volume has been discussed and illustrated. They have pointed out that the disappearance of notch and diastolic peak is caused by the strong and early reflection (backward) wave. Further, with the aid of WPDA, we can also examine phenomena of missing features and ambiguous features with respect to the properties of component waves so as to find a universal approach to tackle these issues.

**Figure 3:**
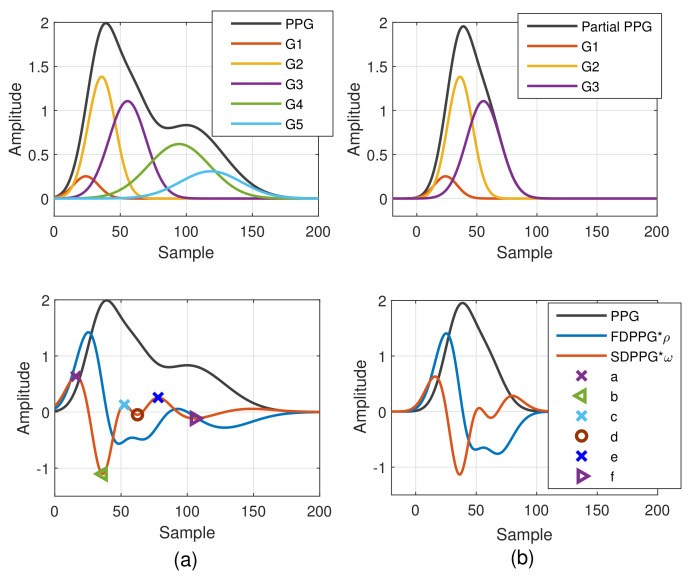
Synthesized PPG with normalized amplitude and its associated first-order derivative PPG, second-order derivative PPG, and five component waves are shown in (**a**). Synthesized PPG excluding the last two component waves (diastolic components) and its first-order derivative PPG and second-order derivative are shown in (**b**).

The Gaussian wave is similar to the pulsatile wave and is widely adopted as the basis wave for PDA [28,31]. Five Gaussian waves were employed here, which are shown to sufficiently support the analysis of the five classes mentioned in Figure 2. Figure 3 first shows a typical PPG pulse of Class 1. The five component waves are also available in the top of Figure 3a. PPG and FDPPG as well as SDPPG are provided in the bottom of Figure 3a with scaling factor ρ and ω. In Figure 3b, the component waves, G4 and G5, are removed. Therefore, the contribution of the first three component waves can be clearly seen. The systolic peak in the PPG waveform, maximal slope in the FDPPG waveform, and a, b, c, as well as d points in the SDPPG waveform, are mainly determined by the component waves in the systole. On the other hand, notch, diastolic peak, and e and f points are influenced by the component waves in the diastole. Note that the widths of component waves in the systole are narrower than those of the component waves in the diastole. Point e is located around the boundary of the systole and diastole.

**Figure 4:**
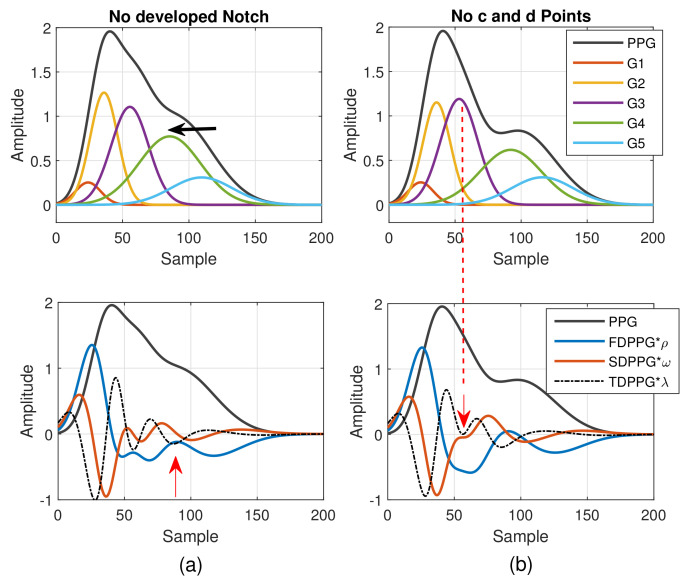
(**a**) Degeneration of notch and diastolic peak in PPG and (**b**) degeneration of c and d points in SDPPG versus the properties of five component waves that constitute synthesized PPG with normalized amplitude and its associated first-order derivative PPG, second-order derivative PPG, and third-order derivative PPG.

As mentioned in [30], different arrival times of the strong and early reflection wave G4 cause changes of the PPG waveforms. Compared to Figure 3a, the early-arrival G4 component wave changes the PPG waveform from Drawber’s Type I, or our Class 1, to Type II that has a horizontal slope. If the peak position of the reflection wave G4 shifts further to the left, the PPG waveform will become Drawber’s Type III that has a monotonically decreased slope and corresponds to our Class 2 as shown in Figure 4a. Note that when there exists developed notch and diastolic peak, the FDPPG intersects with the x-axis and the zero-crossing points correspond to the positions of systolic peak, notch, and diastolic peak. The notch starts to be indistinguishable when the x-axis becomes the tangent to the FDPPG around the red arrow. Furthermore, the local maximum of FDPPG around the red arrow becomes negative while the notch and diastolic peak vanish.

A similar phenomenon can be observed and explained for the disappearance of c and d points in the SDPPG waveform. From Figure 3, we see that the c and d points are generated by the fluctuation of the FDPPG, which is mainly the result of the blood volume increase and decrease introduced by the component waves G2 and G3 in late systole. Hence, if the arrival time and the width of component wave G3 vary with respect to G2, the volume decrease caused by G2 and slight volume increase by G4 may balance the volume changed by G3, which is shown in Figure 4b. The TDPPG waveform is depicted in the figure to see the degeneration of c and d points. In Figure 4b, the c and d points are going to disappear because the x-axis becomes the tangent to TDPPG around the second local minimum. If the second local minimum of TDPPG is a positive value, the SDPPG waveform will lose c and d points (Class 5 in Figure 2e).

**Figure 5:**
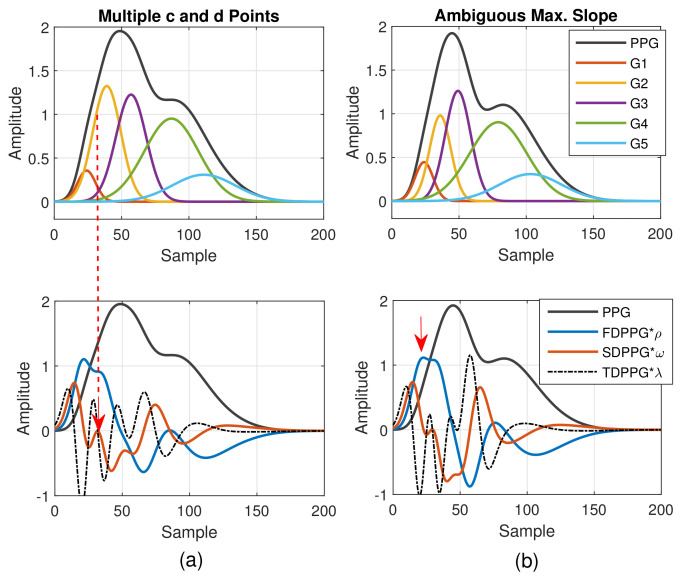
(**a**) Multiple c and d points in SDPPG and (**b**) ambiguous max. slope in FDPPG versus the properties of five component waves that constitute synthesized PPG with normalized amplitude and its associated first-order derivative PPG, second-order derivative PPG, and third-order derivative PPG.

As to the feature ambiguity, the phenomenon of multiple c and d points in Class 4 can be realized in Figure 5. If the component waves G2 and G3 overlap less, then the respective acceleration of blood volume changes caused by G2 and G3 can be observed, which is shown in Figure 5a (Class 4 in Figure 2d). Note that in Table 1, the value of $n_{c}$ is set as the upper bound of the G2 peak position. However, in case of multiple c and d points, the pair of the second local maximum and local minimum is generated mainly due to the second component wave, G2. Hence, in order to set a proper constraint for G2 peak position, it is better to mark the last pair of the local maximum and local minimum before point e as the c and d points to ensure the correct search range.

In Figure 5b, the stronger component wave G3 causes the ambiguous feature of the local maximum in FDPPG. For the ambiguous maximal slope in the ascending segment of the PPG pulse in Class 3, instead of choosing the local maximum with larger amplitude in the early systole, the first local maximum, which could also be an ambiguous feature, such as in Figure 5b, should be identified because in the standard PPG pulse, the maximal slope is mainly caused by the component waves, G1 and G2, instead of G3. If only the maximum in the FDPPG waveform is selected, the position of maximal slope may be unstable and changes alternatively between two possible values in the one-minute recording.

#### 2.2.2. Feature Imputation and Resolving

From the previous discussion, we can realize that missing features and ambiguous features are the result of the variations in intrinsic properties of the component waves. However, their existence causes limitations to the algorithms that can be selected to deal with the PPG-related estimation problems [19] or to the subjects whose PPG signals can be analyzed. Even though the features are missing, the component waves still exist. In [9], the position of a local maximum around the systole boundary in FDPPG was used as an alternative of the diastolic peak position when it vanished. In [11], positions of e and f points in the SDPPG waveform have been suggested when the notch and diastolic peak disappeared. However, no systematic approach for missing and ambiguous features has been proposed to handle our Classes 2 to 5 patterns comprehensively.

To provide effective boundary constraints for WPDA, we propose a missing-feature imputation and ambiguous-feature resolution technique. Note that the positions of the c point and d point gradually become close to each other in the process toward degeneration in Figure 4b. The position of the TDPPG local minimum pointed by the red arrow in Figure 4b effectively indicates the possible upper bound and lower bound of the c and d points. In the case of missing c and d points (Class 5), we then use the position of the second TDPPG local minimum, denoted by $n_{min(2)}^{\left(3\right)}$, for imputed c and d position while the SDPPG amplitude associated with this position is adopted for the imputed c and d amplitude.

(6) $$\left\{\begin{array}{c} n_{c}=n_{d}=n_{min(2)}^{\left(3\right)}\\ A_{n_{c}}^{\left(2\right)}=A_{n_{d}}^{\left(2\right)}=A_{n_{min(2)}^{\left(3\right)}}^{\left(2\right)} \end{array}\right.$$

**Figure 6:**
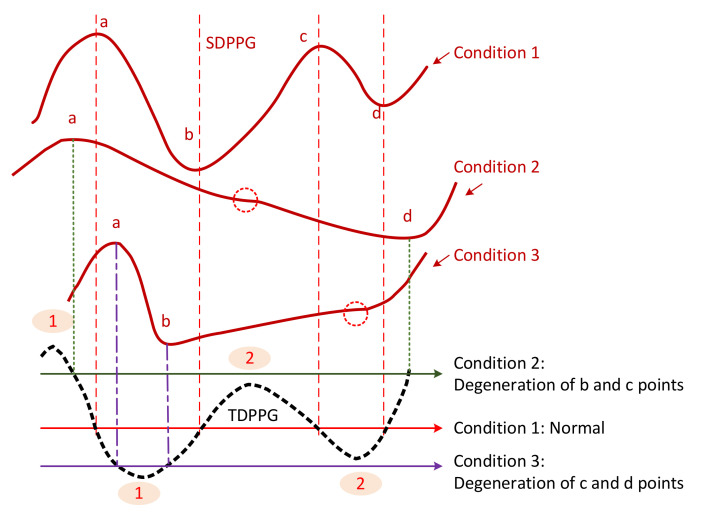
Illustration of feature degeneration.

To generalize the missing-feature imputation concept, we use Figure 6 to illustrate. For the normal condition (condition 1), TDPPG has four zero-crossing points before $n_{e}$. Their positions correspond to a, b, c, and d points of SDPPG. Point b exists when the ascending segment of TDPPG from the first local minimum to the second local maximum intersects with the x-axis. Similarly, points c and d appear when there are zero-crossing points in the next descending and ascending segments of TDPPG. If the second local maximum is smaller than 0 or below the x-axis, such as condition 2, the degeneration of b and c points occurs. On the other hand, if the second local minimum is greater than 0 or above the x-axis, c and d points are missing, such as condition 3.

**Figure 7:**
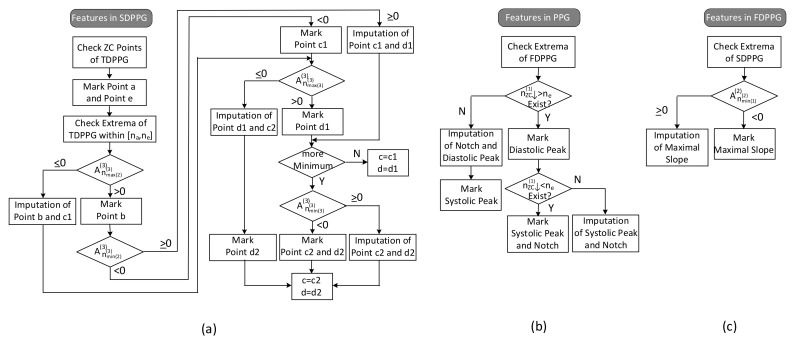
Flow of feature extraction in (**a**) SDPPG, (**b**) PPG, and (**c**) FDPPG.

The feature extraction flow with imputation and ambiguity resolving is described in Figure 7.

(1) Features in SDPPG

Since point e of SDPPG is essential to distinguish features in systole or in diastole, it is first marked. The ventricular systole is about 0.3 s for a 0.8 s cardiac cycle and becomes 0.16 s for a 0.3 s cardic cycle [32]. Note that the duration of diastole decreases more than the duration of systole as the heart rate increases or the R–R interval decreases. Hence, for heart rates below 120 bpm, a window of $\left[0.16+0.1NT_{s},0.3+0.1NT_{s}\right]$ is designated for e-point search, where $NT_{s}$ is the duration of a cardiac cycle. Once point e is determined, point f, which is the next local minimum, and point a, which is the first local maximum, can be identified in SDPPG subsequently. Then, in order to deal with the various conditions for SDPPG features, the extrema of TDPPG between $\left[n_{a},n_{e}\right]$ are checked one by one. Because the multiple c and d points may be accompanied with the feature degeneration condition, we use c1/c2 and d1/d2 to denote the features in the first identification.

The respective values of the second maximum and the second minimum of TDPPG are judged if the degeneration of b and c1 points or the degeneration of c1 and d1 points occurs. Then, the value of the third maximum of TDPPG is verified. If it is the last extremum before $n_{e}$ and is positive, only one pair of c and d points will exist. On the other hand, if there are still extrema before position $n_{e}$ or the third maximum of TDPPG is negative, the case of multiple c and d points will occur. When the third minimum exists, its value decides if the imputation of c2 and d2 points are required or not. In this case, c2 and d2 points are regarded as the desired c and d points.

(2) Features in PPG

If the first local maximum of FDPPG after $n_{e}$, denoted by $A_{n_{max\left(1\right)>n_{e}}^{\left(1\right)}}^{\left(1\right)}$, is negative, then the notch and diastolic peak will disappear. The position of the local maximum [9] in FDPPG and the position of f point [11] have been suggested to be a replacement. Similar to the concept described in Figure 6, a hierarchical imputation is designed for PPG of Class 2 with a single peak. If the position of the first local maximum of FDPPG after $n_{e}$ lies between $n_{e}$ and $n_{f}$, the imputation will be

(7) $$\left\{\begin{array}{c} n_{notch}=n_{dia.}=n_{max\left(1\right)>n_{e}}^{\left(1\right)}\\ A_{n_{notch}}=A_{n_{dia.}}=A_{n_{max\left(1\right)>n_{e}}^{\left(1\right)}} \end{array}\right.$$

Otherwise, the positions of e and f points are selected to be a replacement as suggested in [11],

(8) $$\left\{\begin{array}{c} n_{notch}=n_{e},n_{dia.}=n_{f}\\ A_{n_{notch}}=A_{n_{e}},A_{n_{dia.}}=A_{n_{f}} \end{array}\right.$$

It is also possible that the degeneration features are the systolic peak and notch, especially for wrist PPG, when there is no zero-crossing point in FDPPG before $n_{e}$. In this case, first, if the position of the last local minimum of FDPPG is located between $n_{d}$ and $n_{e}$, the imputation will be

(9) $$\left\{\begin{array}{c} n_{sys.}=n_{notch}=n_{min\left(last\right)<n_{e}}^{\left(1\right)}\\ A_{n_{sys.}}=A_{n_{notch}}=A_{n_{min\left(last\right)<n_{e}}^{\left(1\right)}} \end{array}\right.$$

Otherwise,

(10) $$\left\{\begin{array}{c} n_{notch}=n_{e},n_{sys.}=n_{d}\\ A_{n_{notch}}=A_{n_{e}},A_{n_{sys.}}=A_{n_{d}} \end{array}\right.$$

(3) Features in FDPPG

As to the maximal slope, the first local minimum of SDPPG, $A_{n_{min(1)}^{\left(2\right)}}^{\left(2\right)}$ is examined. If it is negative, then the first zero-crossing point of SDPPG will indicate the maximal slope. Otherwise, the imputed maximal slope (ms) is given by

(11) $$\left\{\begin{array}{c} n_{ms}=n_{min(1)}^{\left(2\right)}\\ A_{n_{ms}}^{\left(1\right)}=A_{n_{min(1)}^{\left(2\right)}}^{\left(1\right)} \end{array}\right.$$

Table 2 summarizes the feature definitions and the positions of imputation, where $n_{ZC↑}^{\left(i\right)}\left(n_{ZC↓}^{\left(i\right)}\right)$ means the zero-crossing point in the ascending(descending) segment of the $i^{th}$-order derivative PPG.

## 3. Results

### 3.1. Statistics of Imputed Features

To verify the effect of imputation and ambiguity resolution, we examined the statistics of the extracted features. A total of 84 subjects were recruited and their ages ranged from 30 to 80 years old. Their systolic blood pressure (SBP) was distributed between 85 and 172 mmHg while the diastolic blood pressure (DBP) was between 50 to 106 mmHg with 44 to 112 bpm. Each subject provided three to five one-minute measurements during the experiment, which was approved by the Research Ethics Committee of National Taiwan University Hospital (No. 201902087RIPA). The wrist and finger PPG signals were captured by SENSIO${}^{\mathrm{TM}}$ and were processed by the flow mentioned previously.

To ensure the correct operations of our algorithm for feature extraction, features of PPG, FDPPG, and SDPPG in 40 one-minute finger PPG recordings from 20 subjects were first checked cycle by cycle for the labeled systolic peak, notch, diastolic peak, maximal slope, and points a to e. The 20 subjects were chosen from the 84 recruited volunteers according to the different skewness values of their finger PPG pulse. A total of 2202 PPG cycles were examined. All features could be fully identified in 2183 (99.14%) of 2202 PPG cycles according to the definitions in Table 2.

**Figure 8:**
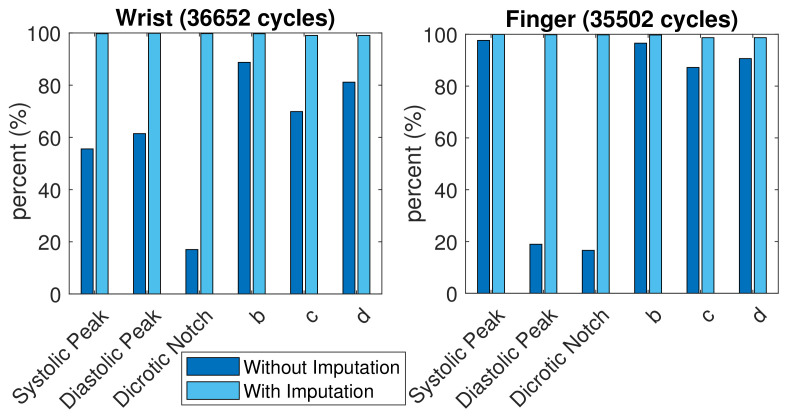
Comparison of feature extraction ratio with and without imputation.

A total of 35,502 finger PPG cycles and 36,652 wrist PPG cycles with qualified SQI were obtained from these subjects. The comparison of successful feature extraction ratio is shown in Figure 8. More c and d points vanished in the wrist PPG cycles than in the finger PPG cycles. The finger PPG pulse with a single peak usually contains the systolic peak while the wrist PPG pulse with a single peak could have vanished systolic peak or vanished diastolic peak. However, with the missing-feature imputation technique, successful feature extraction was achieved in 35,036 (98.7%) of 35,502 finger PPG cycles and 36,307 (99.1%) of 36,652 wrist PPG cycles. When the TDPPG waveform showed no related local maximum (minimum) before $n_{e}$ during imputation, the SDPPG waveform still lacked b and c (c and d) points.

The data processing block in Figure 1 first checked the number of the PPG cycles that had qualified SQI and PD SQI in one-minute recordings. If there have been more than 20 qualified PPG cycles, the recording was regarded as valid and the respective medians of the feature values were calculated for this recording. Denote the median of feature *x* in position $n_{x}$ as ${\bar{n}}_{x}$. There were 418 valid finger PPG recordings and 465 valid wrist PPG recordings without imputation. After imputation and resolving ambiguity, 519 valid finger PPG recordings and 583 valid wrist PPG recordings were acquired.

Table 3 provides the statistics, including median, mean, and standard deviation (std.) of the results from these finger and wrist PPG recordings, respectively. When the imputation did not apply, only the statistics of the recordings without missing values were computed. The number of recordings with missing features is also listed. Because the degeneration involved a pair of features, such as point c and point d or point b and point c, the probability of a missing c point or notch was higher. In addition, when the feature was not resolved and imputed, sometimes, point d could be mistaken as point b and the maximal slope could be unstable. Note that the standard deviation regarding notch in finger PPG is calculated from only 29 recordings. Hence, with the proposed technique, the standard deviations of the feature distributions from these recordings were mostly decreased, especially for the wrist signals. The proposed technique can provide effective and meaningful feature values, which is helpful to the subsequent processing by WPDA and deep learning algorithm.

### 3.2. Coherence between Finger PPG and Wrist PPG

It has been mentioned that higher correlation existed between the finger PAT and PWV than wrist PAT in [16] and more analyzable PPG signals were provided from the finger as well as the earlobe, partially due to rich arterial supply [17]; however, the wrist PPG can be easily acquired from a smart watch. If comprehensive investigation regarding wrist PPG could be conducted, it could markedly improve the applicability of wearable devices in various biomedical scenarios. The intrinsic properties of finger PPG and wrist PPG were then examined and compared in this paper.

**Figure 9:**
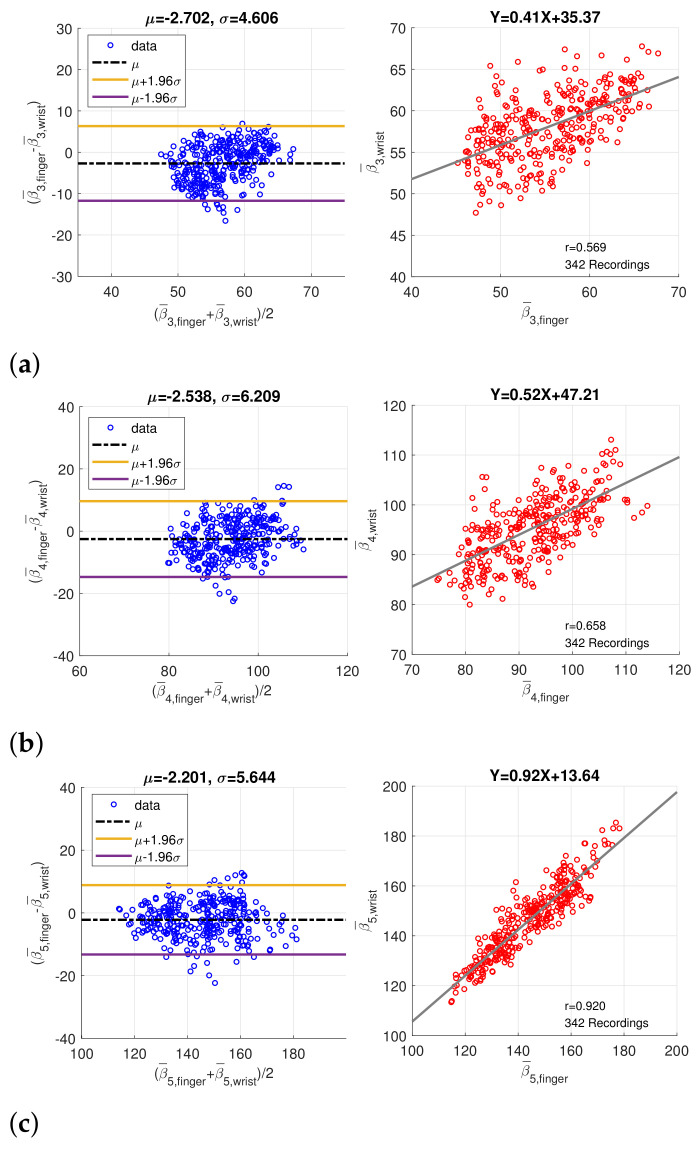
Bland–Altman plots and scatter plots of the positions of (**a**) the 3rd component wave, (**b**) the 4th component wave, and (**c**) the 5th component wave in paired finger PPG and wrist PPG samples.

**Figure 10:**
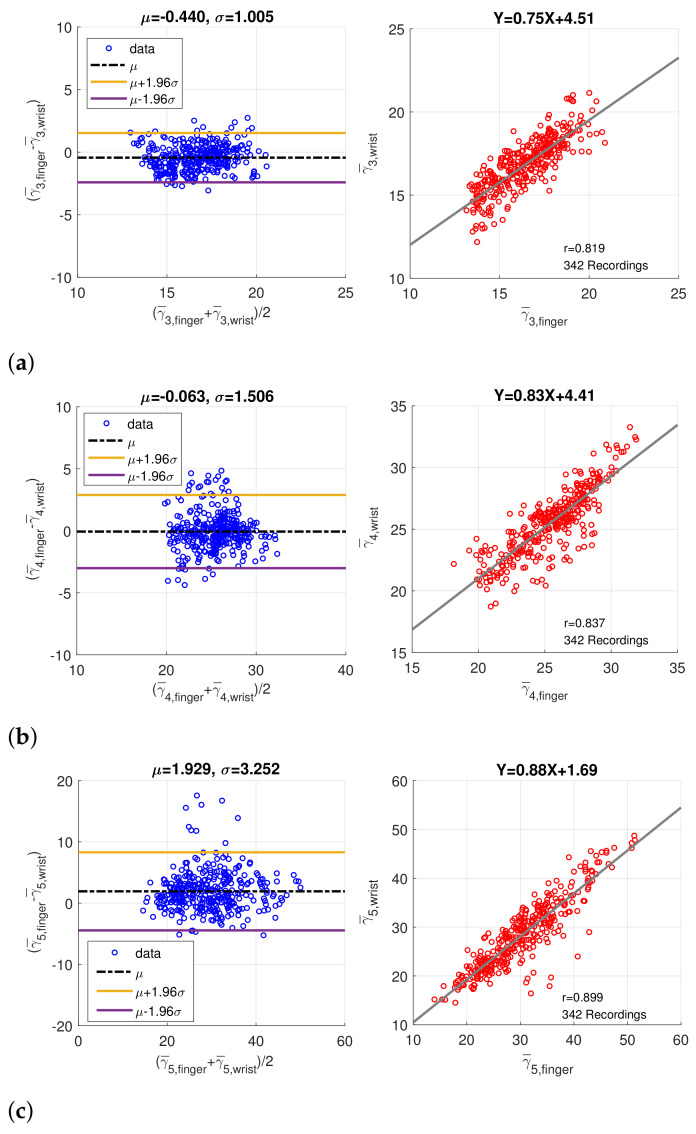
Bland–Altman plots and scatter plots of the width of (**a**) the 3rd component wave, (**b**) the 4th component wave, and (**c**) the 5th component wave in paired finger PPG and wrist PPG samples.

In order to verify the coherence between finger PPG and wrist PPG, only the intersection of the synchronized finger PPG and wrist PPG that were simultaneously qualified by SQI and PD SQI was considered. Similarly, only recordings with more than 20 qualified pulses were regarded as valid, and medians of the amplitude, peak position, and width, denoted by ${\bar{\alpha }}_{i}$, ${\bar{\beta }}_{i}$, and ${\bar{\gamma }}_{i}$, of five component waves were computed. A total of 280 one-minute recordings were acquired from 49 subjects originally. After imputation and resolving ambiguity, we had 342 one-minute recording from 60 subjects. Figure 9 shows the Bland–Altman plots and scatter plots of ${\bar{\beta }}_{3}$, ${\bar{\beta }}_{4}$, and ${\bar{\beta }}_{5}$, the position of component waves G3, G4, and G5, decomposed from the finger and wrist PPG. The mean and standard deviation of the difference in the Bland–Altman plots are denoted as μ and σ. Because the sampling rate is 256Hz, the standard deviations (σ) of differences in Bland–Altman plots of ${\bar{\beta }}_{3}$, ${\bar{\beta }}_{4}$, and ${\bar{\beta }}_{3}$ samples correspond to 17.99 ms, 24.25 ms, and 22.05 ms, respectively. In addition, Figure 10 presents the Bland–Altman plots and scatter plots of the width of the component waves, ${\bar{\gamma }}_{i}$, for i=3,4,5. The standard deviations of differences in Bland–Altman plots are 3.92 ms, 5.87 ms, and 12.70 ms for ${\bar{\gamma }}_{3}$, ${\bar{\gamma }}_{4}$, and ${\bar{\gamma }}_{5}$, respectively. Both the positions and widths of the component waves in the finger PPG pulse and wrist PPG pulse show obvious correlation and coherence. Because the magnitude of each PPG pulse is normalized, the amplitudes of the component waves from the finger PPG pulses and the wrist PPG pulses are not assessed.

In Table 4, the correlation coefficients of the component wave position and width between the finger and wrist PPG cycles are compared to show the improvement by imputation and ambiguity resolution. Higher correlations between the component waves of PPG pulses measured in different sites are revealed if the imputation and ambiguity resolution technique is applied. Define the systolic wave Gs = G1 + G2 + G3 and the diastolic wave Gd = G4 + G5. The forward wave Gf is also given by G1 + G2. The peak positions of the forward wave, systolic wave, and the diastolic wave are indicated by $n_{Gf}$, $n_{Gs}$ and $n_{Gd}$, respectively. According to [29], the stiffness index (SI) can be derived from $n_{Gd}-n_{Gs}$, which is highly related with BP. The correlation coefficient of SI between the finger PPG and wrist PPG becomes 0.543 from 0.324 after the implementation of the proposed technique. Further, $A_{Gd}$ and $A_{Gs}$ are the peak amplitude of the diastolic wave and systolic wave, respectively. The correlation of ratio $A_{Gd}/A_{Gs}$ is 0.391. The peak position of the third component wave to the peak position of the forward wave, defined as $\beta_{3\to Gf}=\beta_{3}-n_{Gf}$, is also shown, which is 0.729. Without feature imputation, only 28 recording pairs are left for valid systolic peak and no recording exists for notch and diastolic peak considering the intersection of wrist and finger PPG results. With the proposed technique, the correlations for notch and diastolic peak between wrist and finger PPG are greater than 0.6. Even though the position of local maximum after point e in SDPPF or point e as well as point f is possibly employed for imputed notch and diastolic peak, the correlation also shows that these features between finger and wrist are consistent to a certain degree.

## 4. Discussion

**Figure 11:**
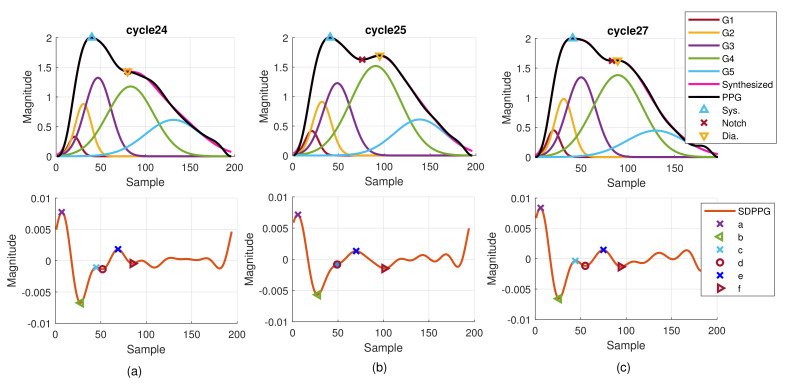
Example of extracted features and decomposed component waves of one subject for (**a**) cycle 24, (**b**) cycle 25, and (**c**) cycle 27.

Although it is well known that the arrival time of strong reflection wave due to the hemodynamic state affects the PPG morphology, the time-varying property can be observed from the PPG cycles in one recording. Figure 11 provides the consecutive finger PPG cycles of one subject. The true PPG pulse is depicted by the blue line with marked systolic peak, notch, and diastolic peak by the program. The decomposed component waves are drawn in the same sub-figure together with the synthesized PPG pulse in magenta color. The SDPPG with extracted features is given in the bottom sub-figure. Points c and d gradually disappear and then appear again. Cycle 26 is similar to cycle 25. It indicates the inherently dynamic nature of the slow-varying component waves. Further, when the amplitude, position, and width of the component waves G2 and G3 changes, it shows that points c and d are influenced. Due to imputation, points c and d in cycle 25 overlap while notch and diastolic peak overlap in cycle 24.

**Figure 12:**
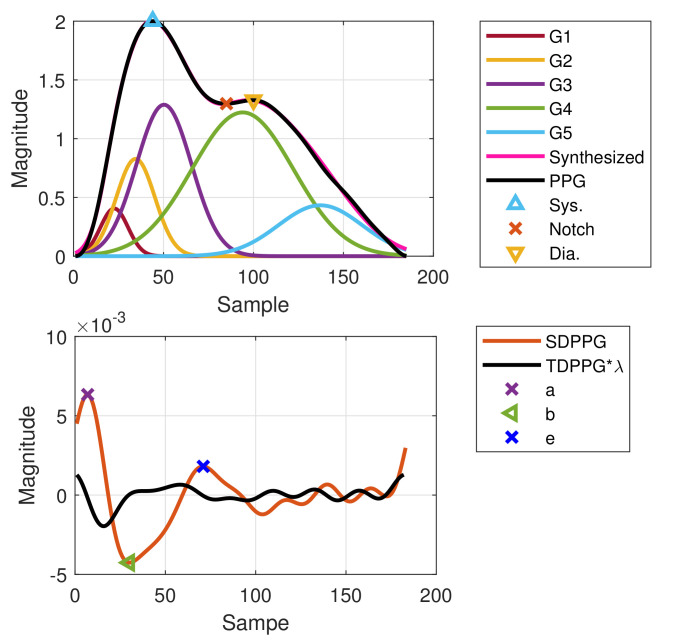
Failed imputation for point c as well as point d but WPDA using positions of points c and d from the previous cycle as a boundary constraint and initial condition.

Even with the imputation technique, the features still cannot be 100% identified. In some cycles, there is no local maximum or local minimum in TDPPG. Thus, the imputation cannot be performed due to the degeneration even in TDPPG. Figure 12 shows one example of failed imputation of points c and d because the TDPPG is monotonically increasing in the region that is supposed to have a local minimum after $n_{b}$. However, owing to the slow-varying property, the positions of points c and d in the previous cycle are employed to set the boundary constraint and initial condition for WPDA of the current cycle, and the component waves G1 to G5 can still be obtained.

**Figure 13:**
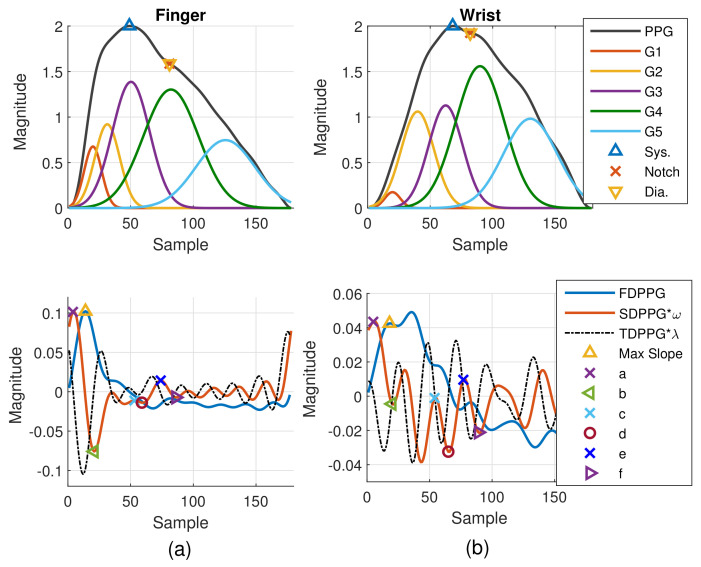
Example of synchronized (**a**) finger and (**b**) wrist PPG cycles with extracted features and decomposed component waves.

One example of synchronized wrist and finger PPG cycles with extracted features and decomposed component waves is given in Figure 13. With resolving feature ambiguity, the extracted features become stable. The proper maximal slope selection in the wrist FDPPG is shown. Although the first local maximum is smaller than the second local maximum, its position is closer to the unambiguous maximal slope in the finger FDPPG. We can see that the stronger reflection waves in the diastole influenced the morphology of the wrist PPG waveform, which has also been mentioned in [17]. The missing-feature imputation also brings the advantage of setting proper boundary constraints for WPDA and thus the component waves can be located correctly.

From the experimental results, although the morphologies of the finger PPG and the wrist PPG are quite different, the intrinsic properties are related. Our proposed feature imputation and ambiguity resolution technique has the following achievements,

- Increaseofavailability: The successful feature extraction ratios are increased for further PPG signal processing.
- Reductionofvariation: The standard deviations of feature distributions are decreased.
- Enhancementofcorrelation: The proper boundary constraints derived from feature extraction can be set and the component waves can be located correctly to enhance the feature correlation.
- Demonstrationofcoherence: Notable coherence of intrinsic properties between wrist and finger PPG exists, especially for the temporal properties.

To the best of the authors’ knowledge, the quantitative comparisons of the finger PPG and wrist PPG component waves have not been studied. Therefore, our proposed imputation and ambiguity resolution technique is essential for using the wrist PPG signals acquired by a smart watch to develop BP, PWV, and vascular age estimations.

In Table 4 and Figure 9 and Figure 10, data of paired finger PPG and wrist PPG are included for evaluation. However, finding the relationship between intra-subject finger PPG and wrist PPG within a given time frame or under specific scenarios is essential to comprehend the applicability of PPG obtained from different measurement sites in daily lives. Motion artifacts and noise often corrupt the signal quality during exercise for subjects wearing a smart watch [33]. A further study can be carried out in these aspects to gain insight into PPG changes caused by various environmental and physical factors, which should be important for the wider applications of smart watches in daily lives.

## 5. Conclusions

Morphology of the finger PPG signals have been widely investigated so as to acquire various features that are highly related with hemodynamic states. Recently, using PPG signals from other measurement sites attracts much attention due to the rapid development of wearable devices. However, feature loss and ambiguity are major limitations for the development of PPG-based algorithms. From the properties of the component waves that constitute the PPG pulse, we then propose a systematic approach to implement missing-feature imputation and ambiguous-feature resolution. The extrema of TDPPG and SDPPG are employed to identify the missing or ambiguous features in the SDPPG and FDPPG waveforms. With the imputation and ambiguity resolution techniques, the feature extraction can become stable with smaller standard deviations and the feature availability from wrist and finger PPG waveforms achieves more than 98.6%. Significant correlations up to 0.92 between finger and wrist PPG signals were revealed for the positions and widths of the third to the fifth component waves. Our proposed algorithm broadens the usage of wrist PPG for BP, PWV, and vascular age estimations.

## Figures and Tables

**Table 1:** Boundary constraints for WPDA [22].

	Amplitude Gain		Peak Position		Wave Width	
*i*	$L_{\alpha_{i}}$	$U_{\alpha_{i}}$	$L_{\beta_{i}}$	$U_{\beta_{i}}$	$L_{\gamma_{i}}$	$U_{\gamma_{i}}$
1	0	$A_{n_{b}}$	$n_{a}$	$n_{b}$	0	$n_{b}/3$
2	0	$A_{n_{sys}}$	$n_{a}$	$n_{c}$	$n_{a}/3$	$n_{d}/3$
3	0	$0.8\times A_{n_{sys}}$	$n_{b}$	$n_{d_{n}}$	$n_{b}/3$	$n_{d_{n}}/3$
4	0	$A_{n_{dias}}$	$n_{f_{n}}$	$(n_{f}+2N)/3$	0	N/3
5	0	$A_{n_{dias}}$	$n_{f}$	*N*	0	N/3

**Table 2:** Summary of feature extraction in normal and imputed conditions. The variable $n_{x}$ denotes the sample position.

Feature	Definition	Normal Condition	Imputed Condition
Systolic peak $\left(n_{sys.}\right)$	PPG peak in systole	$n_{ZC↓}^{\left(1\right)}$ in systole with the largest	1. $n_{min\left(last\right)<n_{e}}^{\left(1\right)}$ if $n_{min\left(last\right)<n_{e}}^{\left(1\right)}\in \left[n_{d},n_{e}\right]$
		PPG amplitude	2. $n_{d}$
Notch $\left(n_{notch}\right)$	PPG local minimum around	$n_{ZC↑}^{\left(1\right)}$	1. Same as imputed systolic peak 1.
	systole boundary		or imputed diastolic peak 1.
			2. $n_{e}$
Diastolic peak $\left(n_{dia.}\right)$	PPG peak in diastole	first $n_{ZC↓}^{\left(1\right)}$ in diastole	1. $n_{max\left(1\right)>n_{e}}^{\left(1\right)}$ if $n_{max\left(1\right)>n_{e}}^{\left(1\right)}\in \left[n_{e},n_{f}\right]$
			2. $n_{f}$
Maximal slope $\left(n_{ms}\right)$	First local maximum in FDPPG	First $n_{ZC↓}^{\left(2\right)}$	$n_{min(1)}^{\left(2\right)}$
Point a $\left(n_{a}\right)$	First local maximum in SDPPG	First $n_{ZC↓}^{\left(3\right)}$	-
Point b $\left(n_{b}\right)$	First local minimum in SDPPG	First $n_{ZC↑}^{\left(3\right)}$	$n_{max(2)}^{\left(3\right)}$
Point c $\left(n_{c}\right)$	Last local maximum in SDPPG	Last $n_{ZC↓}^{\left(3\right)}\in \left[n_{a},n_{e}\right]$	1. $n_{max(2)}^{\left(3\right)}$ if degeneration of b and c1
	before $n_{e}$		2. $n_{min(2)}^{\left(3\right)}$ if degeneration of c1 and d1
			3. $n_{max(3)}^{\left(3\right)}$ if degeneration of d1 and c2
			4. $n_{min(3)}^{\left(3\right)}$ if degeneration of c2 and d2
Point d $\left(n_{d}\right)$	Last local minimum in SDPPG	Last $n_{ZC↑}^{\left(3\right)}\in \left[n_{a},n_{e}\right]$	1. $n_{min(2)}^{\left(3\right)}$ if degeneration of c1 and d1
	before $n_{e}$		2. $n_{max(3)}^{\left(3\right)}$ if degeneration of d1 and c2
			3. $n_{min(3)}^{\left(3\right)}$ if degeneration of c2 and d2
Point e $\left(n_{e}\right)$	Local maximum in SDPPG	$n_{ZC↓}^{\left(3\right)}\in \left[\frac{0.16}{T_{s}}+0.1N,\frac{0.3}{T_{s}}+0.1N\right]$	-
	around end-systolic boundary		
Point f $\left(n_{f}\right)$	First local minimum in SDPPG	First $n_{ZC↑}^{\left(3\right)}$ after $n_{e}$	-
	after $n_{e}$		

**Table 3:** Statistics of features with and without imputation in (**a**) finger PPG recordings and (**b**) wrist PPG recordings.

				(**a**)				
	**Without Imputation & Resolving**				**With Imputation & Resolving**			
	**Total: 418 Recordings**				**Total: 519 Recordings**			
	Median	Mean	Std.	Missing Rec.	Median	Mean	Std.	Missing Rec.
R–R Interval $\left(NT_{s}\right)$ (ms)	841.80	832.75	125.02	0	832.03	824.07	121.01	0
Systolic Peak $\left({\bar{n}}_{sys.}T_{s}\right)$ (ms)	222.65	221.83	42.27	3	222.66	220.31	41.01	0
Notch $\left({\bar{n}}_{notch}T_{s}\right)$ (ms)	328.13	328.86	25.66	389	347.6	346.52	31.52	0
Diastolic Peak $\left({\bar{n}}_{dia.}T_{s}\right)$ (ms)	386.72	385.00	34.96	383	359.38	355.20	31.33	0
Maximal Slope $\left({\bar{n}}_{ms.}T_{s}\right)$ (ms)	70.31	74.25	14.57	0	70.31	70.47	9.29	0
b $\left({\bar{n}}_{b}T_{s}\right)$ (ms)	101.56	104.73	17.97	4	101.56	102.03	12.66	0
c $\left({\bar{n}}_{c}T_{s}\right)$ (ms)	164.06	168.44	27.58	30	167.97	172.62	26.29	0
d $\left({\bar{n}}_{d}T_{s}\right)$ (ms)	222.65	228.05	33.00	20	226.56	228.44	28.79	0
				(**b**)				
	**Without Imputation & Resolving**				**With Imputation & Resolving**			
	**Total: 465 Recordings**				**Total: 583 Recordings**			
	Median	Mean	Std.	Missing Rec.	Median	Mean	Std.	Missing Rec.
R–R Interval $\left(NT_{s}\right)$ (ms)	855.47	862.71	131.58	0	847.66	847.21	131.20	0
Systolic Peak $\left({\bar{n}}_{sys.}T_{s}\right)$ (ms)	281.25	285.47	39.14	220	277.34	273.83	27.70	0
Notch $\left({\bar{n}}_{notch}T_{s}\right)$ (ms)	328.13	331.56	27.77	453	316.41	317.58	22.81	0
Diastolic Peak $\left({\bar{n}}_{dia.}T_{s}\right)$ (ms)	378.91	382.23	33.20	175	371.09	370.00	31.29	0
Maximal Slope $\left({\bar{n}}_{ms.}T_{s}\right)$ (ms)	101.56	106.99	27.07	0	78.13	76.99	12.34	0
b $\left({\bar{n}}_{b}T_{s}\right)$ (ms)	136.72	140.55	35.66	12	89.84	90.46	17.85	0
c $\left({\bar{n}}_{c}T_{s}\right)$ (ms)	187.50	190.27	34.84	116	187.50	186.84	28.59	0
d $\left({\bar{n}}_{d}T_{s}\right)$ (ms)	242.19	242.89	28.98	35	244.14	244.34	21.40	0

**Table 4:** Correlation of the component-wave related properties between the finger PPG and the wrist PPG with and without feature imputation and feature resolving. There are 280 recordings for the results without imputation and 342 recordings for the results with imputation. Correlation coefficients greater than 0.8 are marked by red while correlation coefficients between 0.6 and 0.8 are marked by blue.

	Correlation Coefficient (*p* Value)	
**Feature**	**Without Imputation**	**With Imputation**
Position ${\bar{\beta }}_{3}$	0.573 (*p* < 0.001)	0.569 (*p* < 0.001)
Position ${\bar{\beta }}_{4}$	0.530 (*p* < 0.001)	0.658 (*p* < 0.001)
Position ${\bar{\beta }}_{5}$	0.815 (*p* < 0.001)	0.920 (*p* < 0.001)
Width ${\bar{\gamma }}_{3}$	0.653 (*p* < 0.001)	0.819 (*p* < 0.001)
Width ${\bar{\gamma }}_{4}$	0.715 (*p* < 0.001)	0.837 (*p* < 0.001)
Width ${\bar{\gamma }}_{5}$	0.733 (*p* < 0.001)	0.899 (*p* < 0.001)
Systolic Peak ${\bar{n}}_{sys.}$	0.348 (*p* = 0.070)	0.480 (*p* < 0.001)
Notch ${\bar{n}}_{notch}$	-	0.600 (*p* < 0.001)
Diastolic Peak ${\bar{n}}_{dia.}$	-	0.617 (*p* < 0.001)
SI	0.324 (*p* < 0.001)	0.543 (*p* < 0.001)
$A_{Gd}/A_{Gs}$	0.329 (*p* < 0.001)	0.391 (*p* < 0.001)
${\bar{\beta }}_{3\to Gf}$	0.582 (*p* < 0.001)	0.729 (*p* < 0.001)

## Data Availability

Raw data are available for presentation to the referees and the editors of the journal, if requested.
